# Fetal cardiac magnetic resonance imaging of the descending aorta in suspected left-sided cardiac obstructions

**DOI:** 10.3389/fcvm.2023.1285391

**Published:** 2023-12-01

**Authors:** Katrin Fricke, Daniel Ryd, Constance G. Weismann, Katarina Hanséus, Erik Hedström, Petru Liuba

**Affiliations:** ^1^ Cardiology, Pediatric Heart Center, Skåne University Hospital, Lund, Sweden; ^2^ Pediatrics, Department of Clinical Sciences Lund, Lund University, Lund, Sweden; ^3^ Clinical Physiology, Department of Clinical Sciences Lund, Lund University, Lund, Sweden; ^4^ Department of Clinical Physiology and Nuclear Medicine, Skåne University Hospital, Lund, Sweden; ^5^ Department of Pediatric Cardiology and Pediatric Intensive Care, Ludwig-Maximilian University, Munich, Germany; ^6^ Diagnostic Radiology, Department of Clinical Sciences Lund, Lund University, Lund, Sweden; ^7^ Department of Diagnostic Radiology, Skåne University Hospital, Lund, Sweden

**Keywords:** fetal MRI, fetal diagnosis, left-sided cardiac lesion, HLHS, coarctation of the aorta, critical aortic stenosis

## Abstract

**Background:**

Severe left-sided cardiac obstructions are associated with high morbidity and mortality if not detected in time. The correct prenatal diagnosis of coarctation of the aorta (CoA) is difficult. Fetal cardiac magnetic resonance imaging (CMR) may improve the prenatal diagnosis of complex congenital heart defects. Flow measurements in the ascending aorta could aid in predicting postnatal CoA, but its accurate visualization is challenging.

**Objectives:**

To compare the flow in the descending aorta (DAo) and umbilical vein (UV) in fetuses with suspected left-sided cardiac obstructions with and without the need for postnatal intervention and healthy controls by fetal phase-contrast CMR flow. A second objective was to determine if adding fetal CMR to echocardiography (echo) improves the fetal CoA diagnosis.

**Methods:**

Prospective fetal CMR phase-contrast flow in the DAo and UV and echo studies were conducted between 2017 and 2022.

**Results:**

A total of 46 fetuses with suspected left-sided cardiac obstructions [11 hypoplastic left heart syndrome (HLHS), five critical aortic stenosis (cAS), and 30 CoA] and five controls were included. Neonatal interventions for left-sided cardiac obstructions (*n* = 23) or comfort care (*n* = 1 with HLHS) were pursued in all 16 fetuses with suspected HLHS or cAS and in eight (27%) fetuses with true CoA. DAo or UV flow was not different in fetuses with and without need of intervention. However, DAo and UV flows were lower in fetuses with either retrograde isthmic systolic flow [DAo flow 253 (72) vs. 261 (97) ml/kg/min, *p* = 0.035; UV flow 113 (75) vs. 161 (81) ml/kg/min, *p* = 0.04] or with suspected CoA and restrictive atrial septum [DAo flow 200 (71) vs. 268 (94) ml/kg/min, *p* = 0.04; UV flow 89 vs. 159 (76) ml/kg/min, *p* = 0.04] as well as in those without these changes. Adding fetal CMR to fetal echo predictors for postnatal CoA did not improve the diagnosis of CoA.

**Conclusion:**

Fetal CMR-derived DAo and UV flow measurements do not improve the prenatal diagnosis of left-sided cardiac obstructions, but they could be important in identifying fetuses with a more severe decrease in blood flow across the left side of the heart. The physiological explanation may be a markedly decreased left ventricular cardiac output with subsequent retrograde systolic isthmic flow and decreased total DAo flow.

## Introduction

Severe left-sided cardiac obstructions are associated with high neonatal morbidity and mortality if not detected in fetal life or early postpartum (1, 2). Of these, coarctation of the aorta (CoA) poses a significant challenge in prenatal diagnosis owing to low prenatal detection rates (3) and high false-positive rates up to 75% (4, 5), the latter being associated with parental anxiety and overuse of healthcare-related resources.

Fetal cardiac magnetic resonance imaging (CMR) has the potential to visualize anatomic structures, quantify fetal cardiac blood flow and oxygenation, and measure flow velocities (6–8). Moreover, fetal CMR may help improve prenatal diagnosis of complex congenital heart defects (CHD), especially when acoustic windows are poor (9). Critical left-sided outflow tract obstructions including hypoplastic left heart syndrome (HLHS) and critical aortic stenosis (cAS) exhibit decreased or even reversed flow in the ascending aorta. Even in less severe left-sided obstructive lesions such as aortic arch hypoplasia and CoA, decreased flow in the ascending aorta may be observed. Lloyd et al. suggested that the CMR-based detection of decreased fetal flow in the ascending aorta combined with morphological anomalies of the aortic arch may improve the prenatal diagnosis of CoA (10). However, proper visualization and perpendicular imaging of the ascending aorta by fetal CMR to reliably obtain flow measurements is challenging because of the position and course of the ascending aorta in the fetal thorax and its proximity to neighboring cardiac structures. Quantification of flow in the descending aorta (DAo) by fetal phase-contrast CMR (11) may be an alternative target for more reliable flow measurements. Theoretically, altered flow through abnormal left-sided cardiac structures with increased flow through the right side of the heart and arterial duct may lead to changes in the flow pattern in the descending aorta. In cases with highly decreased left ventricular (LV) cardiac output (CO), retrograde systolic flow in the aortic arch may occur with subsequent changes in total DAo flow. Umbilical venous (UV) flow may be affected by low cardiac output as well as by placental dysfunction (12).

Our earlier retrospective fetal CoA study using echocardiography (echo) identified the carotid-subclavian artery index (CSAI), the product of isthmus-to-duct ratio in the three-vessel trachea view (3VT), and the mitral-to-tricuspid valve ratio (I/D3VTxMV/TV) as reliable predictors of postpartum CoA (13).

The main hypothesis of this study was that fetal CMR may aid in improving detection of fetuses with left-sided cardiac obstruction needing neonatal interventions or at least identifying those with severe left-sided outflow tract obstructions leading to retrograde systolic isthmic flow and subsequent decrease in DAo flow. Furthermore, we hypothesized that *in utero* changes in the DAo flow alone or combined with the previously described echo indices for CoA could further improve the fetal diagnosis of CoA.

Thus, the aims of the present study are as follows:

1) to compare the flow in the DAo and UV
  a) in fetuses with suspected left-sided cardiac obstructions with and without the need of postnatal cardiac intervention and in healthy controls, by fetal phase-contrast CMR; and
  b) in fetuses with retrograde systolic isthmic flow as a possible indirect marker for more severe left-sided cardiac outflow tract obstruction and in fetuses without this flow anomaly;
2) to assess whether combined fetal CMR and echo can improve the fetal diagnosis of CoA needing postnatal intervention.

## Material and methods

This prospective study was conducted at the Children's Heart Center at Skåne University Hospital in Lund, one of two tertiary referral centers for pediatric cardiac surgery in Sweden. Fetuses with echo-based suspicion of left-sided cardiac obstructions and healthy controls were recruited between October 2017 and August 2022.

The following groups were included:

1) fetuses with HLHS;
2) fetuses with critical aortic stenosis (cAS) with or without CoA;
3) fetuses with suspected CoA, with or without aortic arch hypoplasia, mild mitral valve stenosis, and ventricular septal defects (VSDs); and
4) healthy controls.

The fetuses were further categorized as follows:

1) fetuses with left-sided cardiac obstructions in need of postpartum intervention vs. all others;
2) fetuses with retrograde systolic isthmic flow (as a possible indirect sign of severe left-sided cardiac outflow tract obstruction) vs. all others; and
3) fetuses with suspected CoA needing postpartum CoA intervention (true CoA) vs. those not requiring postpartum intervention (false positive CoA).

The study was approved by the Regional Ethical Review Board (2017/175). All pregnant women gave written informed consent before participation.

### Fetal echocardiography measurements

All anatomic measurements were conducted by an experienced fetal cardiologist (KF) utilizing PACS (Sectra, Linköping, Sweden) or SyngoDynamics (Siemens, Germany). The following ratios predictive for postpartum CoA in our prior retrospective fetal echocardiography study (13) were computed: (1) aortic isthmus-to-arterial duct (I/D) in the three-vessel trachea view (3VT) in isolation and (2) as a product with the mitral-to-tricuspid valve ratio (I/D3VTxMV/TV), and (3) the CSAI, the ratio of the transverse aortic arch diameter prior to the take-off of the left subclavian artery to the distance between the left carotid and the left subclavian artery (14). The following was documented: flow direction across the aortic arch and the interatrial communication, the appearance of the atrial septum (normal, redundant, restrictive, intact), flow velocity in the aortic isthmus, presence of a contraductal shelf, the maximum diameter of the DAo at the diaphragmal level, and the existence of additional cardiac anomalies. Retrograde isthmic flow was defined as isthmic flow reversal in systole, whereas reversed end-systolic or diastolic flow was designated as bidirectional isthmic flow. A restrictive atrial septum in fetuses with suspected CoA was defined as an interatrial communication with hardly any right-to-left shunt, quantitatively assessed by color Doppler. In fetuses with HLHS or cAS, a highly restrictive atrial septum was defined as an antegrade to retrograde pulmonary venous velocity time integral (VTI) ratio lower than 3 (15).

### Fetal CMR measurements

Phase-contrast fetal CMR at 1.5 T Aera (Siemens, Erlangen Germany), carried out in non-breath-hold and gated by Doppler ultrasound (smart-sync; Northh Medical, Hamburg, Germany), was used to quantify blood flow in the DAo at the level of the diaphragm and in the intraabdominal part of the umbilical vein (16). Smart-sync-gated flow measurements were normalized for fetal weight assessed by three-dimensional (3D) CMR (17). Indexed total, peak, and minimum blood flows, as peak and mean velocities in the DAo, were measured. Upslope and downslope were calculated for all flow profiles in the DAo and normalized for net flow. All image analyses were done using the Segment version 3.3 (Medviso AB, Lund, Sweden) (18). Two observers (KF and DR) conducted the flow measurements independently. Data were anonymized and both observers were blinded to postpartum outcome.

Inter- and intraobserver as well as inter-modality reliability were assessed by applying Bland–Altman plots and/or the intraclass correlation coefficient (ICC). Cases with divergent results were jointly reviewed by KF and EH.

### Postpartum approach and outcome

All fetuses with suspicion of HLHS, cAS, or CoA were planned for delivery at our tertiary center, ensuring prompt access to pediatric cardiac surgery. High-risk pregnancies [HLHS or cAS with restrictive atrial septum (RAS)/intact atrial septum (IAS)] were delivered by cesarean section to facilitate emergency postnatal intervention. Prostaglandins were administered in all neonates with HLHS and those with cAS and signs of a duct-dependent systemic circulation.

Newborns with prenatally suspected CoA underwent several echocardiograms within the first week of life. If CoA was not demonstrated at the first postpartum echocardiogram, the newborn was monitored carefully, and prostaglandins were only administered if clinical or echocardiographic signs of CoA emerged. In individual cases with pronounced hypoplasia of the aortic arch or aortic isthmus, prostaglandins were administered instantly and continued until surgical repair. Cases with prenatally suspected CoA and the need of surgical CoA repair within the neonatal period were designated as true positives, all others, as false positives.

Both fetal growth restriction (FGR) and small for gestational age (SGA) were defined as an estimated fetal or birth weight below the 10th percentile for the gestational age.

### Statistics

Data are presented as median and interquartile range (IQR). For group-wise comparison of continuous variables, we applied either the Mann–Whitney *U*-test or the Kruskal–Wallis test. Categorical variables were compared by using the Chi-square or Fisher's exact test. ICC with 95% confidence intervals were provided. The result was rated as excellent when an ICC of >0.90 with corresponding confidence interval was reached. ICCs between 0.75 and 0.90 indicated good reliability, between 0.50 and 0.75 indicated moderate reliability, and <0.50 indicated poor reliability (19). Hazard ratios (95% confidence interval) were applied for the multivariate analysis. A *p*-value of <0.05 was considered statistically significant, whereas *p*-values of >0.05 and <0.10 were considered a trend. The Statistical Package for Social Sciences version 28 (IBM SPSS, Chicago, IL, USA) was used.

## Results

A total of 49 women with fetuses with suspected left-sided cardiac obstructions and five healthy controls agreed to participate in the present study. Three cases in the group with suspected left-sided obstructions were excluded owing to suboptimal flow examinations in the DAo and UV. Of the remaining 51 fetuses, 30 had suspected CoA [of which eight (27%) developed CoA with the need of neonatal repair—true CoA], 11 had HLHS, five, cAS, and further five were healthy controls. Aside from one neonate with HLHS and IAS, managed according to comfort care, all patients with HLHS and cAS underwent cardiac surgery in the neonatal period.

The main characteristics of the study cohort are shown in Table 1. The median gestational age at fetal CMR was 35.5 (3.5) weeks with an estimated fetal weight of 2,720 (1,157) g. FGR was encountered in seven fetuses at the time of fetal examination. The fetal echocardiogram and CMR were performed in succession [median 0 (0.3) days]. No intrauterine deaths occurred. None of the healthy controls or patients with suspected CoA died during follow-up. Five infants with HLHS or cAS died in the neonatal period and one with HLHS and partial anomalous pulmonary venous drainage (PAPVD) in the first year of life. Altogether 24 infants underwent neonatal cardiac surgery at the median age of 5 (4) days. Of these, one newborn with borderline left ventricle underwent solely a surgical ductal closure at 5 days of age. One additional infant had VSD repair at the age of 1 year.

**Table 1:** Main characteristics.

	Median [IQR] or *n* (%)	*n*
Fetal cardiac examination		
Gestational age (weeks)	35.5 [3.5]	51
Estimated weight by MR (g)	2,720 [1,157]	51
Fetal growth restriction	7 (14)	51
Postpartum outcome		
Gestational age (weeks)	39.6 [1.9]	51
Weight (g)	3,340 [612]	51
Small for gestational age	7 (14)	51
Female sex	20 (41)	49
Neonatal cardiac surgery	24 (47)^a^	51
HLHS	10 (91)^b^	11
cAS	5 (100)	5
True CoA	8 (100)	8
End-to-end	2 (25)	8
Extended aortic arch repair	6 (75)	8
Age at operation (days)	5 [4]	24
Neonatal death	5 (10)	51
One-year mortality	6 (12)	51

PDA, patent ductus arteriosus.

Data are presented as median [IQR] or *n* (%).

^a^ One false positive had a PDA closure at 5 days of age.

^b^ One newborn with HLHS/IAS was deemed unsuitable for univentricular palliation.

### Fetal CMR flow measurements related to outcome

**Figure 1:**
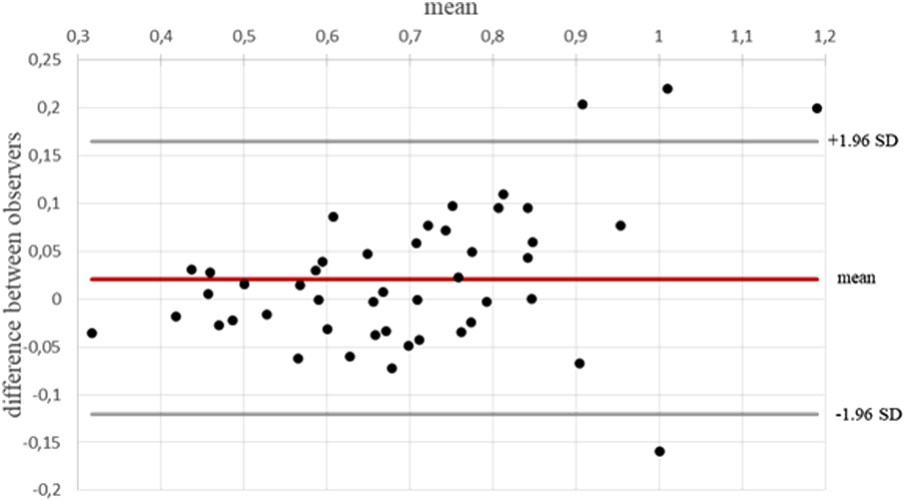
Interobserver reliability of blood flow measurements in the DAo. Bland–Altman plot and ICC (0.95) indicating excellent interobserver reliability.

**Figure 2:**
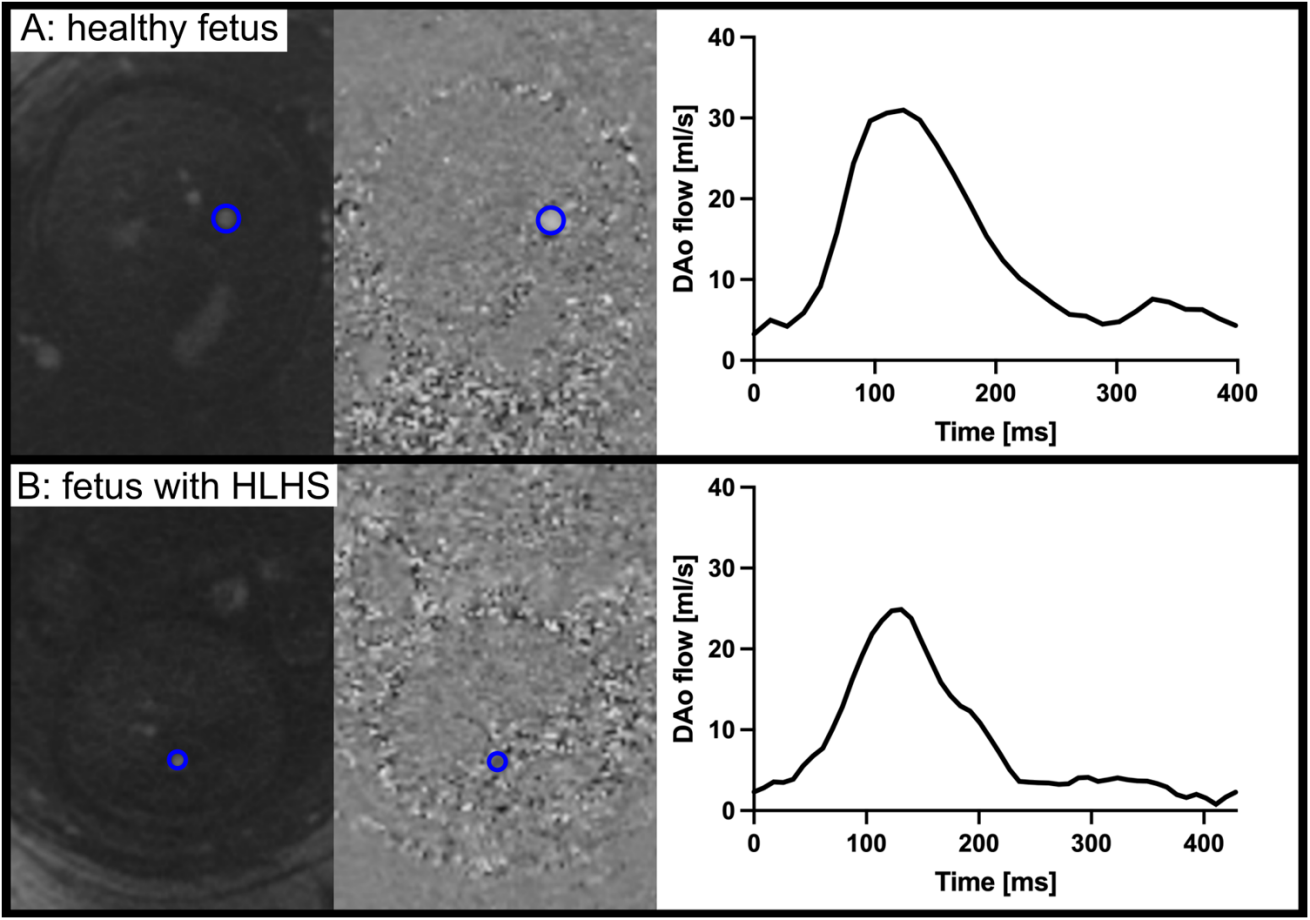
Examples of fetal CMR phase-contrast images and corresponding descending aorta flow curves in a healthy fetus (**A**) and in a fetus with HLHS (**B**). Although similar in heart rate and gestational age, the DAo flow volume is larger in the healthy fetus. Note that data shown are in absolute flow and not normalized to fetal weight.

There was excellent agreement in fetal CMR DAo flow measurements between Observers 1 (KF) and 2 (DR); [Bland–Altman plot, Figure 1; ICC 0.95 (0.92–0.97)]. Figure 2 shows examples of fetal CMR phase-contrast images and corresponding DAo flow curves in a healthy fetus and in a fetus with HLHS.

FGR (*p* > 0.4), need of neonatal cardiac surgery (*p* > 0.2), neonatal death (*p* > 0.6), and 1-year mortality (*p* > 0.7) were not associated with changes in DAo and UV flows (data not shown).

There were no differences in DAo or UV flows between fetuses with left-sided cardiac obstructions and healthy fetuses based on postpartum management (Table 2).

**Table 2:** Left-sided cardiac obstructions with need of neonatal repair vs. all others.

	False positives and healthy controls	*n*	Left-sided cardiac obstructions	*n*	*p*
Fetal cardiac examination					
Gestational age (weeks)	36.1 [3.2]	27	35.2 [4.7]	24	0.4
Estimated weight by MR (g)	2,754 [977]	27	2,579 [934]	24	0.3
Fetal growth restriction	4 (15)	27	3 (13)	24	1
Associated cardiac anomalies					
L-SVC	1 (5)	22	1 (4)	24	1
VSD	3 (14)	22	4 (17)	24	1
RAS	2 (9)	22	5 (21)	24	0.4
Fetal echocardiogram					
Foramen ovale flow					
Right–left	13 (59)	22	1 (4)	24	**<0** **.** **001**
Bidirectional	9 (41)		10 (42)		
Left–right	0 (0)		9 (38)		
None	0 (0)		4 (17)		
Retrograde systolic isthmic flow	2 (7)	27	17 (71)	24	**<0** **.** **001**
Fetal CMR					
Descending aorta flow					
Indexed total flow (ml/kg/min)	255 [97]	25	253 [80]	22	0.3
Indexed peak flow (ml/kg/s)	11.5 [2.4]	25	11.2 [4.5]	22	0.5
Indexed minimum flow (ml/kg/s)	0.7 [1.3]	25	0.7 [1.2]	22	0.5
Quotient minimum flow/total flow	0.2 [0.3]	25	0.2 [2.8]	22	0.5
Upslope/net flow (ml/s)	0.032 [0.01]	25	0.034 [0.01]	22	0.9
Downslope/net flow (ml/s)	−0.018 [0.01]	25	−0.017 [0.01]	22	0.8
Indexed net flow (ml/kg)	1.0 [0.4]	25	0.9 [0.5]	22	0.4
Mean max velocity (cm/s)	67.3 [16.4]	25	69.4 [17.5]	22	0.2
Mean min velocity (cm/s)	7.5 [5.2]	25	7.7 [8.6]	22	0.7
Mean max–mean min velocity (cm/s)	57.2 [16.4]	25	62.1 [18.4]	22	0.2
Umbilical vein flow (ml/kg/min)	142 [93]	20	136 [69]	16	0.8
DAo/UV flow	2.0 [0.8]	18	1.9 [0.9]	14	0.7
Postpartum outcome					
Gestational age (weeks)	39.6 [1.9]	27	39.4 [2.1]	24	0.8
Weight (g)	3,340 [612]	27	3,394 [711]	24	0.8
Small for gestational age	3 (11)	27	4 (17)	24	0.7
Female sex	11 (44)	25	9 (38)	24	0.8
Neonatal cardiac surgery	1 (4)^a^	27	23 (96)^b^	24	**<0** **.** **001**
Age at operation (days)	5 [—]	2	5 [4]	23	0.8
Neonatal death	0 (0)	27	5 (21)	24	**0** **.** **01**
One-year mortality	0 (0)	27	6 (25)	24	**0** **.** **006**

L-SVC, persistent left superior vena cava; RAS, restrictive atrial septum.

Data are presented as median [IQR] or *n* (%).

^a^ PDA closure.

^b^ One newborn with HLHS/IAS was deemed unsuitable for univentricular palliation.

Bold values indicate significant *p*-values.

#### Association between retrograde systolic isthmic flow on fetal echo with fetal CMR flows and postpartum outcome

**Figure 3:**
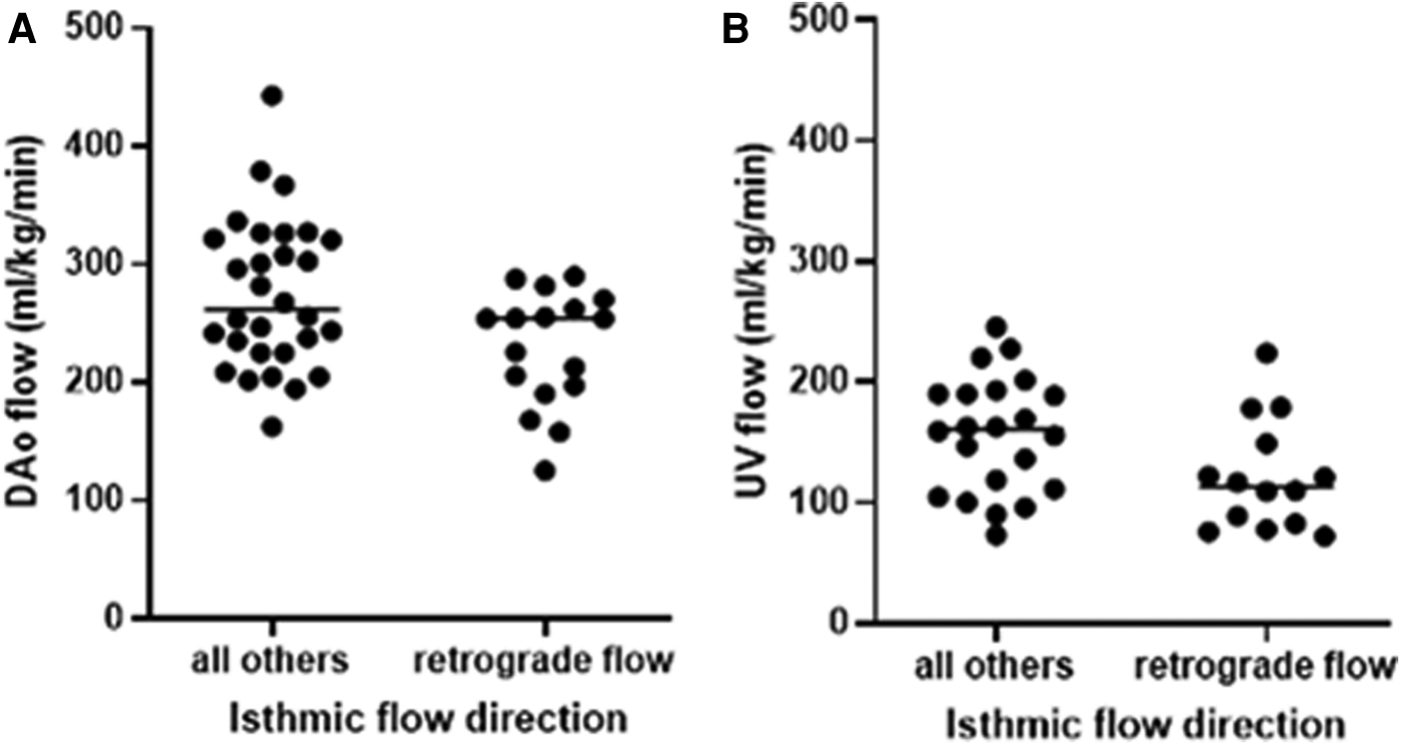
Blood flow in the (**A**) fetal DAo and (**B**) UV in presence or absence of retrograde isthmic flow with significantly decreased flows when retrograde flow in the aortic isthmus was present. Solid lines indicate median.

All 11 fetuses with HLHS, four (80%) with cAS, two (25%) with true CoA, and two (9%) false positives exhibited retrograde systolic flow in the aortic isthmus. All patients with HLHS and cAS and fetal retrograde systolic isthmic flow had a duct-dependent systemic circulation, requiring intravenous administration of prostaglandins. Retrograde systolic isthmic flow was associated with decreased total DAo and UV flow, DAo net flow, and changes in the DAo downslope flow pattern (Table 3, Figure 3). There was a trend to decreased DAo minimum flow (*p* = 0.09). Retrograde isthmic flow was also associated with neonatal cardiac surgery (*p* < 0.001) and 1-year mortality (*p* = 0.02) (Table 3).

**Table 3:** Fetuses with retrograde systolic isthmic flow vs. all other fetuses.

	All other fetuses	*n*	Retrograde systolic isthmic flow	*n*	*p*
Fetal cardiac examination					
Gestational age (weeks)	35.1 [3.9]	32	36.6 [3.9]	19	0.09
Estimated weight by MR (g)	2,627 [1,032]	32	2,820 [1,076]	19	0.3
Fetal growth restriction	4 (13)	32	3 (16)	19	1
Associated cardiac anomalies					
L-SVC	2 (7)	27	0 (0)	19	0.3
VSD	5 (19)	27	2 (11)	19	0.6
Atrial septal aneurysm	11 (41)	27	0 (0)	19	**0** **.** **001**
RAS	1 (4)	27	6 (32)	19	**0** **.** **02**
Fetal echocardiogram					
Foramen ovale flow					
Right–left	12 (44)	27	2 (11)	19	**<0** **.** **001**
Bidirectional	14 (52)		5 (26)		
Left–right	0 (0)		9 (47)		
None	1 (4)		3 (16)		
Fetal CMR					
Descending aorta flow					
Indexed total flow (ml/kg/min)	261 [97]	30	253 [72]	17	**0** **.** **035**
Indexed peak flow (ml/kg/s)	11.6 [2.7]	30	10.9 [4.0]	17	0.2
Indexed minimum flow (ml/kg/s)	0.9 [1.3]	30	0.5 [1.1]	17	0.09
Quotient minimum flow/total flow	0.2 [0.3]	30	0.1 [0.3]	17	0.1
Upslope/net flow (ml/s)	0.032 [0.1]	30	0.038 [0.01]	17	0.2
Downslope/net flow (ml/s)	−0.016 [0.01]	30	−0.021 [0.01]	17	**0** **.** **03**
Indexed net flow (ml/kg)	1.3 [0.4]	30	0.8 [0.3]	17	**0** **.** **03**
Mean max velocity (cm/s)	67.8 [15.5]	30	70.7 [20]	17	0.3
Mean min velocity (cm/s)	7.8 [5.2]	30	7.5 [5.8]	17	0.3
Mean max–mean min velocity	57.5 [14.8]	30	66.2 [23.6]	17	0.1
Umbilical vein flow					
Total flow (ml/kg/min)	161 [81]	22	113 [75]	14	**0** **.** **04**
DAo/UV flow	1.8 [0.7]	20	2.2 [0.7]	12	0.17
Postpartum outcome					
Gestational age (weeks)	39.6 [1.7]	32	39.1 [2.2]	19	0.7
Weight (g)	3,421 [729]	32	3,284 [593]	19	0.3
Small for gestational age	3 (9)	32	4 (21)	19	0.4
Female sex	13 (43)	30	7 (37)	19	0.8
Neonatal cardiac operation	7 (22)	32	17 (89)	19	**<0** **.** **001**
Age at operation (days)	5 [7]	7	5 [3]	17	0.4
Neonatal death	1 (3)^a^	32	4 (21)	19	**0** **.** **058**
One-year mortality	1 (3)^a^	32	5 (26)	19	**0** **.** **02**

Data are presented as median [IQR] or *n* (%).

^a^ newborn with critical aortic stenosis dying because of metabolic disease.

Bold values indicate significant *p*-values.

#### Fetal CMR flow variables in the fetus with HLHS or cAS

Both fetuses with HLHS or cAS exhibited decreased DAo peak flows [10.5 (2.7) ml/kg/s vs. 11.9 (3.2) ml/kg/s; *p* = 0.04] and a trend to decreased total DAo flows [247 (74) ml/kg/min vs. 255 (105) ml/kg/min; *p* = 0.08] compared to fetuses of all other subgroups. Furthermore, DAo net flows were lowest in this group with significant differences between fetuses with cAS and healthy controls [0.85 (0.2) vs. 1.0 (0.3) ml/kg; *p* = 0.01]. A trend to decreased minimum DAo velocities [7.5 (7.5) vs. 10.1 (5.4); *p* = 0.09] and a larger difference between minimum and maximum DAo velocities [90 (13.1) vs. 86 (4.7) %; *p* = 0.07] was noted in fetuses with HLHS compared to healthy controls. The DAo flow pattern did not differ between HLHS or cAS and all other included fetuses (*p* > 0.3) (data not shown). The presence of RAS/IAS in fetuses with HLHS or cAS was not associated with a further decrease in DAo and UV flows (*p* > 0.3) (data not shown) but was associated with both neonatal (60% vs. 0%; *p* = 0.02) and 1-year mortality (50% vs. 0%, *p* = 0.04).

#### Differences in fetal echo and CMR variables between true CoA vs. false positives

**Figure 4:**
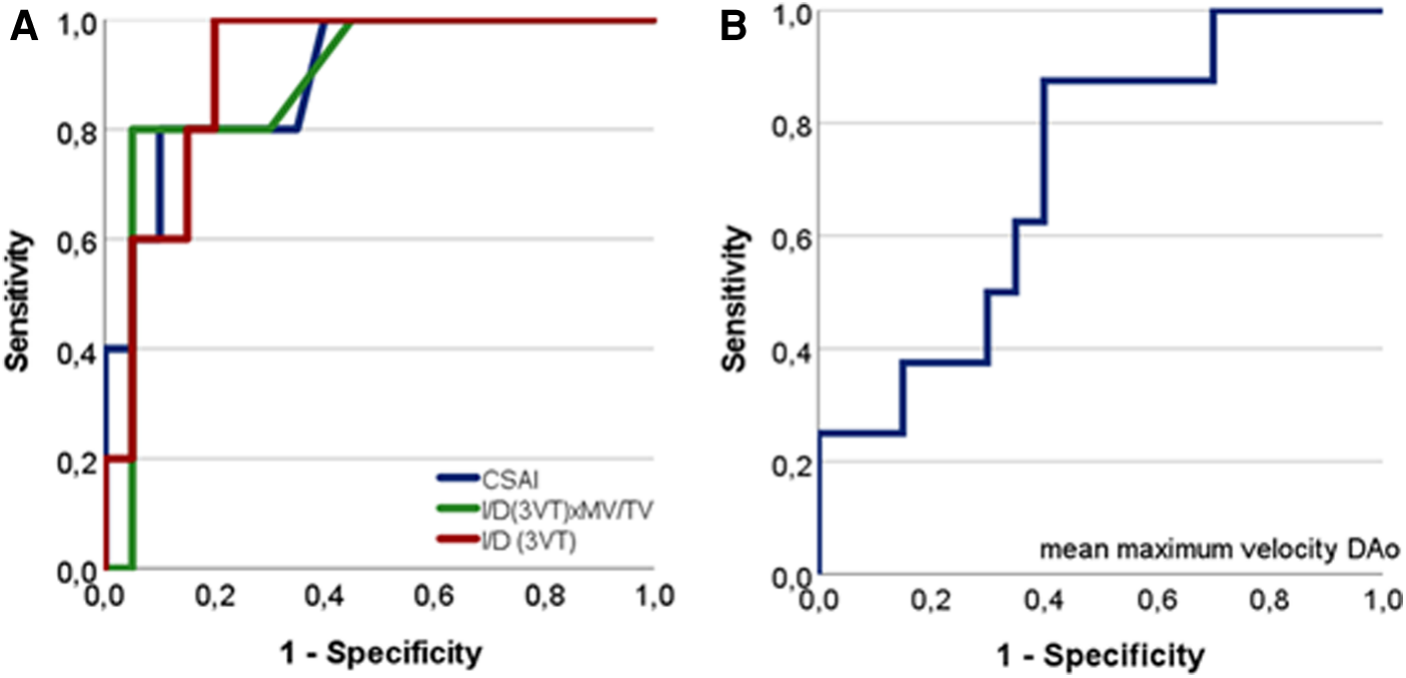
Receiver operating characteristic (ROC) curves relating fetal CMR and echo-derived variables to outcome true CoA. (**A**) Fetal echo variables related to outcome true CoA predicting true CoA with high accuracy. CSAI (AUC = 0.89; *p* = 0.007); I/D(3VT): diameter isthmus aorta to arterial duct in the three-vessel trachea view (AUC = 0.91; *p* = 0.005); I/D(3VT)xMV/TV: product of I/D(3VT) with the mitral-to-tricuspid valve ratio (AUC = 0.89; *p* = 0.009). (**B**) Fetal CMR measured mean maximum velocity related to outcome true CoA (AUC 0.71; *p* = 0.08).

The previously identified echocardiographic ratios [I/D(3VT); I/D(3VT)xMV/TV; CSAI] (13), presence of a contraductal shelf, and flow anomalies across the foramen ovale were linked to true CoA (Table 4; Figure 4A). Associated cardiac defects, RAS, and retrograde systolic isthmic flows were equally distributed in both groups. However, fetuses with true CoA were examined earlier in gestation than false positives (Table 4). Fetal CMR DAo and UV flows were not linked to postpartum outcome. Yet, a trend to increased DAo maximum velocities in fetuses with true CoA was observed (Table 4, Figure 4B). When combining fetal echocardiographic ratios linked to true CoA with fetal CMR flow data in the multivariate regression analysis, no positive association between DAo or UV flow data and postpartum outcome was noted (*p* ≥ 0.2) (data not shown).

**Table 4:** True CoA vs. false positives.

	False positive	*n*	True positive	*n*	*p*
Fetal cardiac examination					
Gestational age (weeks)	36.2 [3.3]	22	33.3 [4.1]	8	**0** **.** **02**
Estimated weight by MR (g)	2,737 [1,016]	22	2,134 [432]	8	**0** **.** **02**
Fetal growth restriction	2 (9)	22	1 (13)	8	1
Associated cardiac anomalies					
L-SVC	1 (5)	22	1 (13)	8	0.5
VSD	3 (14)	22	3 (38)	8	0.3
Mitral valve anomaly	0 (0)	22	1 (13)	8	0.3
Restrictive atrial septum	2 (9)	22	2 (25)	8	0.3
Fetal echocardiogram					
I/D (3VT)	0.67 [0.12]	22	0.39 [0.18]	8	**<0** **.** **001**
I/D3VTxMV/TV	0.43 [0.08]	22	0.25 [0.17]	8	**<0** **.** **001**
Flow direction across foramen ovale					
Right–left	13 (59)	22	1 (13)	8	**0** **.** **03**
Bidirectional	9 (41)		6 (75)		
No flow	0 (0)		1 (13)		
Flow direction isthmus aorta					
Antegrade flow	9 (41)	22	3 (38)	8	0.5
Bidirectional flow	11 (50)		3 (38)		
Retrograde systolic flow	2 (9)		2 (25)		
CSAI	1.0 [0.5]	20	0.67 [0.27]	5	**0** **.** **004**
Contraductal shelf	7 (32)	22	7 (88)	8	**0** **.** **01**
Isthmal displacement/DAo diameter	0.00 [0.28]	22	0.44 [0.53]	8	**0** **.** **005**
Fetal CMR					
Descending aorta flow					
Total flow (ml/kg/min)	248 [100]	20	268 [141]	8	0.5
Indexed peak flow (ml/kg/s)	11.4 [2.5]	20	13.5 [3.8]	8	0.2
Indexed minimum flow (ml/kg/s)	0.8 [1.3]	20	0.7 [1.8]	8	0.6
Quotient minimum flow/total flow	0.2 [0.3]	20	0.1 [0.5]	8	0.5
Upslope/net flow (ml/s)	0.037 [0.01]	20	0.036 [0.02]	8	0.9
Downslope/net flow (ml/s)	−0.02 [0.01]	20	−0.01 [0.03]	8	0.6
Indexed net flow (ml/kg)	1.0 [0.5]	20	1.1 [0.6]	8	0.6
Mean max velocity (cm/s)	66.9 [16.9]	20	72.0 [15.8]	8	**0** **.** **08**
Mean min velocity (cm/s)	6.8 [5.6]	20	8.2 [5.9]	8	0.76
Mean max–mean min velocity (cm/s)	57.8 [19.7]	20	59.8 [19.5]	8	0.2
Umbilical vein flow (ml/kg/min)	142 [93]	16	163 [80]	5	0.2
DAo/UV flow	2.0 [0.6]	14	1.7 [1.0]	5	0.7
Postpartum outcome					
Gestational age (weeks)	39.3 [1.7]	22	40.2 [1.6]	8	0.3
Weight (g)	3,387 [628]	22	3,728 [902]	8	0.2
Small for gestational age	2 (9)	22	1 (13)	8	1
Female sex	11 (55)	20	4 (50)	8	1
Age at CoA repair (days)	—	22	7 [9]	8	

I, isthmus, D, arterial duct; MV, mitral valve; TV, tricuspid valve.

Data are presented as median [IQR] or *n* (%).

Bold values indicate significant *p*-values.

A restrictive atrial septum in both false positive CoA and with true CoA (*n* = 4) was linked to decreased total DAo and UV flow, minimum DAo flow, DAo net flow, and changes in the DAo flow pattern (upslope and downslope). In addition, higher maximum DAo velocity and the DAo/UV flow quote were noted (Table 5). Three out of four of these fetuses had retrograde systolic flow in the aortic isthmus; none had FGR.

**Table 5:** Restrictive atrial septum in the fetuses with suspected CoA.

	No RAS	*n*	RAS	*n*	*p*
Fetal cardiac examination					
Gestational age (weeks)	35.1 [4.0]	26	38.4 [4.7]	4	0.08
Estimated weight by MR (g)	2,552 [803]	26	3,535 [986]	4	0.2
Fetal growth restriction	3 (12)	26	0 (0)	4	1
Associated cardiac anomalies					
L-SVC	2 (8)	26	0 (0)	4	0.7
VSD	6 (23)	26	0 (0)	4	0.6
Mitral valve anomaly	0 (0)	26	1 (25)	4	0.1
Fetal echocardiogram					
Retrograde systolic isthmic flow	1 (4)	26	3 (75)	4	**0** **.** **004**
Isthmal displacement/DAo diameter	0.00 [0.36]	26	0.47 [1.4]	4	0.09
Fetal CMR					
Descending aorta flow					
Total flow (ml/kg/min)	268 [94]	24	200 [71]	4	**0** **.** **048**
Indexed peak flow (ml/kg/s)	11.5 [3.3]	24	12.5 [3.6]	4	0.6
Indexed minimum flow (ml/kg/s)	1.1 [1.3]	24	−0.3 [2.4]	4	**0** **.** **007**
Quotient minimum flow/total flow	0.2 [0.3]	24	−0.1 [0.7]	4	**0** **.** **006**
Upslope/net flow (ml/s)	0.033 [0.01]	24	0.04 [0.02]	4	**0** **.** **03**
Downslope/net flow (ml/s)	−0.016 [0.01]	24	−0.032 [0.02]	4	**0** **.** **036**
Indexed net flow (ml/kg)	1.1 [0.4]	24	0.7 [0.1]	4	**0** **.** **01**
Mean max velocity (cm/s)	65.5 [14.7]	24	84.5 [7.4]	4	**0** **.** **009**
Mean min velocity (cm/s)	7.8 [5.4]	24	5.03 [12.3]	4	0.1
Mean max–mean min velocity (cm/s)	57.5 [15.5]	24	77.8 [21.5]	4	**0** **.** **01**
Umbilical vein flow					
Total flow (ml/kg/min)	159 [76]	18	89 [—]	3	**0** **.** **035**
DAo/UV flow	1.7 [0.6]	16	2.3 [—]	3	**0** **.** **03**
Postpartum outcome					
Gestational age (weeks)	39.3 [1.6]	26	40.3 [0.7]	4	**0** **.** **04**
Weight (g)	3,237 [658]	26	3,771 [872]	4	0.08
Small for gestational age	3 (12)	26	0 (0)	4	1
Female sex	14 (54)	26	1 (25)	4	0.6

Data are presented as median [IQR] or *n* (%).

Bold values indicate significant *p*-values.

## Discussion

Our findings indicate that fetal CMR flow measurements in the DAo and UV do not improve the overall detection of fetuses needing neonatal intervention for left-sided cardiac obstructions including HLHS, cAS, and CoA. However, there were marked changes in the DAo and UV flow in the presence of systolic retrograde flow in the aortic isthmus of fetuses with severe left ventricular outflow obstructions or RAS in fetuses with suspected CoA, suggesting that flow assessment in the DAo by CMR may provide useful additional information in detecting fetuses with severely impaired flow through the left side of the heart.

### Fetal CMR flow measurements as predictors of left-sided cardiac obstruction

In this study, DAo and UV flow characteristics of fetuses with postnatal left-sided cardiac obstructions in need of neonatal interventions were not different from those in healthy newborns including false positives and controls. Lloyd et al. have previously shown that postnatal development of CoA was associated with decreased CMR flow in the fetal ascending aorta (10). In general, the technically achievable spatial resolution on fetal CMR may be inadequate to reliably assess ascending aortic flow. Hence, we chose to focus on DAo flow, which could be reliably measured as evidenced by excellent interobserver reliability. Similarly, Lloyd et al. reported that visualization of the ascending aorta is technically difficult as reflected by poor interobserver reliability for ascending aortic two-dimensional measurements (10). However, interobserver reliability for ascending aortic flow measurements was not provided, even though fetal ascending aortic flow was identified as an important predictor of postpartum CoA (10).

The absence of differences in DAo flow between fetuses with left-sided cardiac obstructions and healthy ones may be explained by different flow physiology depending on the degree of obstruction. Fetuses with more severe left-sided cardiac obstructions and retrograde systolic isthmic flow may exhibit decreased combined cardiac output and total DAo flow, whereas those with less severe left-sided cardiac obstructions and subsequently decreased but antegrade or bidirectional isthmic flow may exhibit similar total DAo flows due to a compensatory increase in flow through the right side of the heart and the arterial duct.

#### Association between retrograde systolic isthmic flow on fetal echo with fetal CMR flows and postpartum outcome

In this study, 90% of fetuses with retrograde systolic isthmic flow had severe left-sided outflow tract obstruction (100% with HLHS, 80% with cAS, and 25% with true CoA). There was an association between retrograde systolic isthmic flow and decreased total and net flow in the DAo, changes in the DAo flow pattern, and decreased UV flow.

Our finding of retrograde systolic isthmic flow as a predictor of critical left-sided obstructive lesions is consistent with prior studies. Isthmic flow reversal may balance the right- and left-sided cardiac volumes in the fetus (20). While brief flow reversal in end-systole or diastole may occur in healthy fetuses during late gestation because of increasing right ventricular (RV) dominance (20, 21), systolic retrograde flow appears to be more specific to critical left-sided obstructive lesions (20), preponderantly in HLHS (65%), followed by AS (8%), CoA (5%), but also in RAS (5%) (20). Computational fluid dynamics (CFD) simulation and fetal echo Doppler recordings mirror our findings: a gradual decrease in the ascending aortic blood flow leads initially to isthmic flow reversal in end-systole, followed by diastole, and ultimately, when the aortic blood flow is reduced significantly to less than 20%–40%, throughout systole (20, 21).

A previous fetal CMR study showed a decrease in combined cardiac output by 20% in fetuses with left-sided cardiac outflow tract obstructions including fetuses with HLHS and cAS and/or CoA (22). This could explain the decreased flow in the DAo in the presence of retrograde flow through the aortic isthmus compensating for the severely decreased antegrade to retrograde flow in the ascending aorta. Abnormal RV function appears to be another cause of the decreased combined cardiac output in fetuses with HLHS or cAS with evolving HLHS (23, 24). Diastolic RV dysfunction (24–26), with a compensatory increase in RV size, systolic function, and cardiac output (25, 27, 28), is seen in fetuses with HLHS. However, these changes may not fully compensate for the hypoplastic or malfunctioning LV (25). RV dysfunction may be more pronounced in fetuses with cAS with evolving HLHS (23). In our cohort, three out of five fetuses with cAS exhibited a moderately to severely decreased RV function, with the dilated, hypertensive LV likely impeding the RV filling. Hence, both the LV and RV dysfunctions in fetuses with HLHS and cAS may lead to decreased combined CO and subsequent DAo flow.

Four fetuses with suspected CoA (two true CoA, two false positives) had retrograde systolic flow in the aortic isthmus. In the two fetuses with true CoA and one false positive CoA, there were echo signs of marked underdevelopment of the left-sided cardiac structures including the aortic arch. The two with true CoA underwent extended aortic arch reconstruction in the neonatal period as an indirect sign of a severe aortic arch obstruction. One of these two also had a significant mitral valve stenosis, which required mitral valve repair.

Furthermore, three out of four fetuses with suspected CoA had RAS, which was associated with decreased minimum and total DAo flows, UV flows, changes in the DAo flow pattern, and retrograde systolic flow in the aortic isthmus. Two of them did not develop CoA postpartum. A patent foramen ovale facilitates intrauterine LV filling by unhindered streaming of oxygen-rich blood to the left side of the heart and brain (29). RAS may induce decreased LV filling and cardiac output, retrograde aortic arch flow, and subsequent decrease of total DAo flow. Interestingly, only a minority of fetuses with a borderline left heart due to RAS develop CoA postpartum, which agrees with our data (30). The reason for the association between RAS in fetuses with suspected CoA and decreased UV flow is uncertain. Decreased DAo flows may lead to diminished blood return to the placenta and consecutively decreased UV flow. Alternatively, decreased UV flow may lead to decreased blood flow streaming across the foramen ovale, decreased LV filling, ascending aorta, and DAo flows.

In this study, one fetus with true CoA and a borderline LV with marked aortic arch hypoplasia and retrograde systolic isthmic flow also had FGR. An earlier study indicated a link between FGR and lower fetal aorta velocities and umbilical flows per placental weight (31). In the current study, FGR was not associated with decreased flow in the DAo or UV. In FGR, diastolic but not systolic isthmic flow reversal is present (32, 33), suggesting that the pronounced outflow tract obstruction rather than the FGR was the reason for the observed systolic isthmic flow reversal in this patient.

#### Discrimination between true CoA and false positives with help of fetal CMR

While the current study validated previously reported fetal echo predictors of postpartum CoA (13), CMR did not add additional information when evaluating fetuses with suspected CoA regarding postpartum outcome. The trend to increased DAo mean maximum velocity, observed in fetuses with true CoA, did not reach significant levels in the multivariate analysis. Furthermore, fetuses with postpartum confirmation of CoA had a lower gestational age and fetal weight at the time of the fetal CMR. Despite flow data being adjusted for fetal weight, an impact of gestational age on fetal flows cannot be ruled out.

Lloyd et al. suggested a link between decreased CMR-derived flow in the fetal ascending aorta and aortic isthmus and morphological changes in the aortic isthmus and their association with postnatal CoA diagnosis. Negative mean isthmic flows in fetuses with true CoA were quoted (10). Flows in the aortic isthmus were indirectly calculated by subtracting the superior vena cava (SVC) from ascending aorta flows (10). It is possible that the associated cardiac defects such as VSDs, atrioventricular septal defect (AVSDs), and mitral valve anomalies, which were predominantly found in fetuses with true CoA unlike false positives, may have led to a further decrease in the ascending aorta and isthmic flow in fetuses with true CoA in Lloyd et al. (10).

In our study, there was no difference in the isthmic flow direction between fetuses with true CoA and false positives. This is in agreement with other studies that reported that bidirectional isthmic flow or isthmic flow reversal has a low sensitivity and specificity in detecting CoA prenatally (13, 34–36). Furthermore, not only the degree but also the site of the isthmic narrowing may play a role in the occurrence of fetal systolic isthmic flow reversal (37). Furthermore, the definitive narrowing in the aortic isthmus may only occur in the neonatal period when the arterial duct closes (22). In these cases, no obvious isthmic flow deviation occurs *in utero*.

Decreased ascending aortic flow, presumably more pronounced in fetuses with true CoA rather than false positives, will be balanced by a flow increase through the right heart and arterial duct, resulting in the same amount of DAo flow. No differences in the DAo flow pattern between fetuses with true CoA and false positives were observed in our study, probably because the slight difference in left ventricular output decrease and right ventricular output increase did not have the same significant impact on the DAo flow pattern as the retrograde systolic isthmic flow.

## Limitations

Our study was limited by its small subgroup sample size, making subgroup comparison difficult. Furthermore, the small number of cases with true CoA and the difference in gestational age between true CoA and false positives at the time of the fetal CMR examination could have confounded our results and obscured potential differences between these groups. Data regarding placental weight were not available.

## Conclusions

Fetal CMR flow measurements in the DAo and UV do not improve the prenatal overall diagnosis of left-sided cardiac obstructions in need of neonatal intervention including fetuses with suspected CoA. However, its implementation may provide useful additional information in detecting fetuses with severely impaired flow through the left side of the heart including those with retrograde systolic isthmic flow in HLHS, cAS, or CoA with severe arch hypoplasia, especially when the acoustic fetal echo windows are poor. The physiological explanation is likely a marked decrease in left ventricular cardiac output with subsequent retrograde systolic isthmic flow and decreased DAo flow.

## Data Availability

The raw data supporting the conclusions of this article will be made available by the authors, without undue reservation.
